# Serological and Molecular Characterization of Occult HBV Infection in Blood Donors from South Italy

**DOI:** 10.3390/v16010071

**Published:** 2023-12-31

**Authors:** Alessia Sticchi Damiani, Vera Holzmayer, Claudio Galli, Mariangela De Nuzzo, Mark Anderson, Gavin Cloherty, Nicola Di Renzo

**Affiliations:** 1 Servizio Immunotrasfusionale, A.O. Vito Fazzi, 73100 Lecce, Italy; mari.denuzzo@libero.it (M.D.N.); direnzo.ematolecce@gmail.com (N.D.R.); 2 R&D, Abbott Diagnostics, Chicago, IL 60064, USA; vera.holzmayer@abbott.com (V.H.); mark.anderson6@abbott.com (M.A.); gavin.cloherty@abbott.com (G.C.); 3 Independent Researcher, 00139 Roma, Italy; claudiogalli26@gmail.com

**Keywords:** hepatitis B virus, occult infection, blood donation, HBV-DNA, HBsAg, DNA sequencing, viral mutants

## Abstract

Despite good vaccine coverage and careful blood donor selection policies, hepatitis B virus (HBV) is still the most frequent viral infection among blood donors (BDs) in Italy, mostly in the occult form (OBI). We studied the virological features of OBI in BDs from South Italy by serology, molecular testing for HBV-DNA, and sequencing for HBV genotypes and mutations. One hundred and two samples from 95 BDs (22.1% first time, 87.9% regular, median age 57 years) positive for HBV-DNA and negative for HBsAg were retrospectively analyzed. HBV biomarkers were detected in 96.9% (anti-HBc in 44.2%, anti-HBc plus anti-HBs in 49.5%, anti-HBs alone in 3.2%). No risk factor was declared by 45.3% of donors. HBV-DNA levels were very low (median: 7 IU/mL). All samples harbored HBV genotype D and single or multiple mutations in the S gene were found in 28/36 sequences analyzed and in 75% of donors. Mutations were unrelated to gender, donor group or serological patterns. An HBsAg assay with enhanced sensitivity was positive in samples from seven donors (7.4%), two of which negative for HBV-DNA by real-time PCR. OBI still represents a risk for HBV transmission from blood donations; screening by highly sensitive serological and molecular assays is warranted.

## 1. Introduction

The first consensus definition of an occult hepatitis B infection (OBI) has been established in 2009 [1] as the detection of replication-competent hepatitis B virus (HBV) DNA in the liver and/or in the blood in subjects that test negative for the HBV surface antigen (HBsAg). The understanding of OBI has evolved from a rare phenomenon [2] to a natural evolution over the course of HBV infection [3]. This stems from the evidence that the replicative cycle of HBV encompasses the production of a replication-competent viral DNA reservoir (covalently closed circular DNA, or ccc-DNA) that is harbored in the nucleus of infected cells and sustains viral replication that persists indefinitely after recovery from an acute infection and the apparent establishment of an immunity towards HBV [3,4]. Since the introduction of HBV-DNA screening on blood donation with the aim of increasing blood safety [5], a considerable number of donors have been found positive for HBV-DNA at low levels while testing negative for HBsAg, thus fulfilling the definition of OBI. The percentage of those finding ranges from less than 1% to 10% or more [5,6,7,8,9] depending on the sensitivity of the assays employed to detect HBV-DNA [10] and mainly on HBV endemicity in different areas [6,7,8,9]. In Italy, universal vaccination against HBV started in 1991 for all infants, with an estimated coverage around 95% [11] but HBV prevalence is still intermediate, especially in the southern regions, in middle aged and elderly populations that are not included in the vaccine cohorts [12]. In this study, we aimed to characterize the serological patterns and molecular aspects of OBI in a cohort of volunteer blood donors from Apulia, a region of South Italy where screening blood donations for HBV-DNA started in 2008.

## 2. Materials and Methods

This study has been carried out on repository surplus plasma specimens obtained from volunteer blood donors over the years 2018–2020. In that time frame, over 220,000 donations have been collected at different sites across South Apulia (Salento) and biological validation by mandatory testing for HBsAg and HBV-DNA is centralized at the validation center of the blood transfusion service at ‘Vito Fazzi’ hospital in Lecce. As an additional precautionary measure as well as for epidemiological purposes, all donations from first-time donors are also tested for antibodies towards the hepatitis B core antigen (anti-HBc), which represents the most reliable marker for a current or past HBV infection [13]. The use of surplus specimens for research purposes has been authorized by the ethics committee of the ‘Vito Fazzi’ hospital whenever samples are completely anonymized and cannot not be traced back to the individual donors and that dataset for any study involving those samples will not be shared publicly. Therefore, the only demographic information available for this study are age, gender, donor status (regular or first time) and potential risk factors for HBV infection collected through a standard pre-donation questionnaire.

### 2.1. Serological and Molecular Testing

All samples were negative upon routine screening for HBsAg (ARCHITECT HBsAg II, Abbott GmbH, Wiesbaden, Germany, declared sensitivity 0.05 IU/mL) and positive for anti-HBc (ARCHITECT) and HBV-DNA by an assay with a declared sensitivity of 2 IU/mL (Cobas Multiplex HIV, HCV & HBV nucleic acid test, Roche Diagnostics AG, Rotkreuz, Switzerland). Residual plasma specimens were stored at −80 °C, shipped to Abbott R&D and further tested for anti-HBc to confirm the initial positive result, for antibodies towards HBsAg (anti-HBs) by commercially available chemiluminescent methods (CMIA) on the ARCHITECT platform (Abbott Core Laboratory, Abbott Park, IL, USA) and for HBsAg by a CMIA assay with enhanced sensitivity (ARCHITECT HBsAg NEXT qualitative, Abbott). This is a fully automated CMIA with an improved analytical sensitivity of 0.0052 IU/mL [14]. HBV DNA levels were quantitated with Abbott RealTime HBV (LLOQ 10 IU/mL) (Abbott Molecular, Des Plaines, IL, USA). 

### 2.2. HBV Gene Sequencing

HBV DNA was extracted from 0.5 mL of plasma using an automated protocol DNA-protK-500-50 (research use only) on the m2000sp system (Abbott Molecular). First- and second-round PCR were performed to amplify the preS1-S region using Amplitaq Gold^®^ DNA polymerase (Applied Biosystems, Foster City, CA, USA). First-round primers were HBV2813 F (5′-TCATTTTGTGGGTCACCATATT-3′, nt 2811–2832) and 18R (5′−CCCATGAAGTTTAGGGAATAAC-3′, nt 860–881); second-round primers were HBV-2822 F (5′-GGGTCACCAT ATTCTTGGGAAC-3′, nt 2820–2841) and 19R (5′-GTTAGGGTTTAAATGTATACCC-3′, nt 822–843) amplifying a 1245 base pair fragment. The 50 μL PCR reaction (first and second rounds) contained 0.4 μM of each primer, 2.5 mM MgCl_2_, 0.8 μM dNTP mix and 25 μL extracted DNA for first-round PCR, or 2 μL of first-round PCR as template for the second round. First- and second-round amplifications consisted of preincubation at 95 °C (10 min), 40 cycles at 95 °C (20 s), annealing at 50 °C (45 s), extension at 66 °C (2.5 min for the first round or 1.5 min for the second round), and final extension at 72 °C (10 min). Both strands of purified amplification products were sequenced directly using the BigDye^®^ Terminator v3.1 Cycle Sequencing kit and the ABI 3500xl Genetic Analyzer (Applied Biosystems, Foster City, CA, USA). Sequence data were assembled and edited using Sequencher software (version 5.4.6; Gene Codes Corporation, Ann Arbor, MI, USA). Positions with sequence ambiguities were assigned the appropriate IUPAC designation. Genotype was determined by phylogenetic analysis using the PHYLIP v3.5c software package (J. Felsenstein, University of Washington, Seattle, Washington). Nucleotide sequences were aligned with the reference sequences representing genotypes A–I using BioEdit 7.0.4.1 [14,15].

### 2.3. Statistical Analysis

Descriptive results are presented as the medians when variables are non-normally distributed, and as a percent (%) for categorical variables. Differences in categorical variables between groups were evaluated with Pearson’s chi-squared or Fisher’s exact test according to sample size, with a significant difference being ≤0.05.

## 3. Results

### 3.1. Study Population

**Figure 1:**
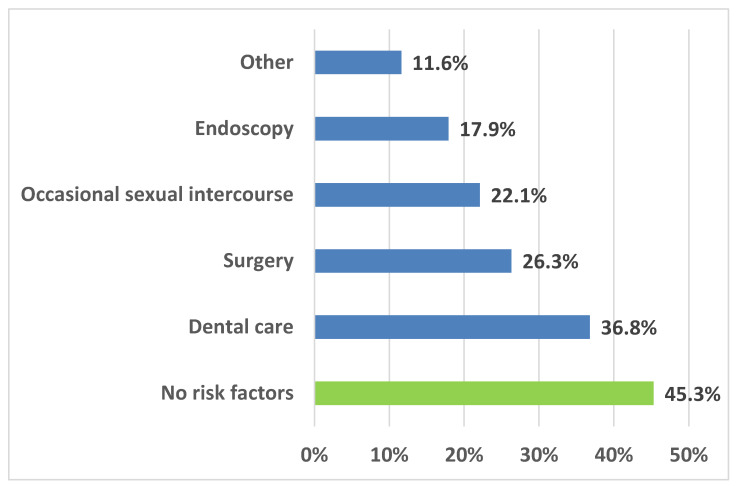
Distribution of risk factors for hepatitis B virus infection among 95 volunteer blood donors from South Italy. The total exceeds 100% because more than one risk factor has been declared by 34 donors (35.8%).

We have retrospectively analyzed routine samples obtained after biological validation from 95 Italian volunteer blood donors. Of those, 21 were first-time donors (FT: 17 males, 4 females, median age 57 years, range 36–67) and 74 were regular donors (R: 62 males, 12 females, median age 58 years, range 34–70). Ninety-two donors (94.7%) were aged >43 years and thus were not included in the population cohorts included in the mass vaccination program against hepatitis B [11], with no significant differences between genders or donor type (Table 1). The risk factors most frequently linked to HBV infection [11,12], except vertical transmission, have been explored by a standard pre-donation questionnaire. Thirty-four donors (35.8%) reported more than one risk factor, the most frequent ones being those related to medical interventions (dental care, surgery, endoscopy), summing up to 81%, while 43 donors (45.3%) did not declare any risk factor. (Figure 1). The only significant difference was the frequency of occasional sexual intercourse, reported by 37.5% of females vs. 19.0% of males (*p* < 0.05).

### 3.2. Serological Results

A total of 102 surplus plasma samples were available from those 95 donors: 1 sample from 92 donors, 4 samples from 2 donors 2 two samples from 1 donor. After testing for HBV serological biomarkers, a positive result for anti-HBc was confirmed in 89 out of 95 donors (93.7%). Of those, 47 (49.5%) were also positive for anti-HBs while 42 (44.2%) were positive only for anti-HBc. Three donors (3.2%) were positive only for anti-HBs, with levels ranging from 14.2 to 98.2 mIU/mL, and three (3.2%) had no HBV antibody biomarkers. The differences in the frequency of the various patterns for HBV markers were not significant between genders neither according to donor type, regular of first-time. Testing by the HBsAg Next assay positively identified seven donors (7.4%, all males, 2 FT and 5 R, median age 54 years), of which three were also positive for anti-HBs (one with values > 1000 mIU/mL) (Table 1). 

### 3.3. Molecular Biology

By real-time HBV-DNA, only 28 samples (29.5%) were positive for HBV-DNA with a median level of 7 IU/mL (range: 1.15–35 IU/mL). A positive result by Pre-S1/S PCR was found in 36 of 98 samples tested, obtained from 91 donors, giving a positive rate among donors of 30.8%, with no significant differences between genders, donor type or serological patterns of HBV biomarkers; four samples from as many donors could not be tested due to insufficient volume. All 36 samples with a positive Pre-S1/S PCR result were sequenced and all were genotype D (D3 in 29 cases, D4 in 5, D1 in 1, D2 in 1). Single or multiple mutations in the S region were found in 28/36 samples (73.1%), corresponding to 21 out of 28 donors (75%) whose samples were positive by the research PCR assay. In four samples, both escape mutations and a mutation linked to resistance to antiviral treatment (amino acid positions 169 and 173) were identified (Table 2 and Supplemental Figure S1). Among the escape mutations, those in the V1 loop of the *a* determinant of HBsAg.

**Figure 2:**
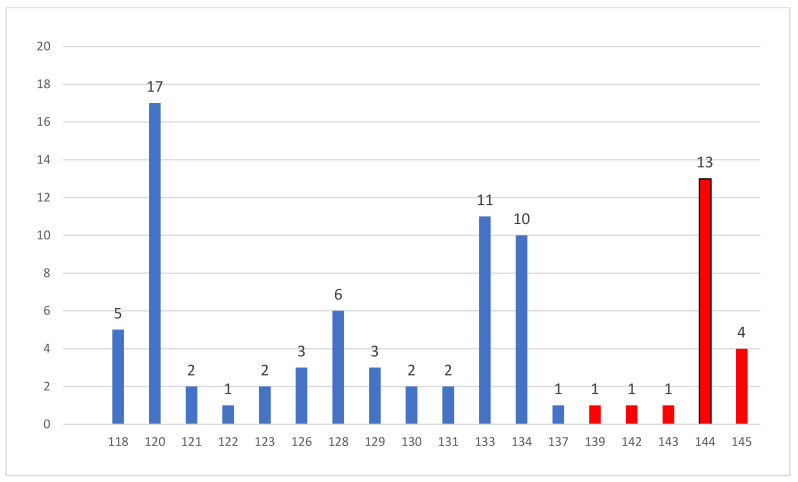
Number of mutations in the *a* determinant of the S gene region of the HBV genome identified after amplification of the Pre-S1/S region of HBV DNA in 36 samples obtained from 91 blood donors. Mutations in the V1 loop in blue, mutations in the V2 loop in red.

Amino acids 107–138 were most frequent (65 out of 87 total mutations, or 74.7%), and 20 (23%) were found in the V2 loop (amino acids 139–147), with only 2 outside the *a* determinant portion of the S-coding region (Figure 2). On the three donors from whom multiple specimens were available, we identified several mutations and the simultaneous presence of 2–4 HBV strains. Two HBV strains, one with three escape mutations and the other one with two drug resistance mutations, were found in another donor and multiple mutations were found in 11 other donors (Table 2). The frequency of HBsAg mutations was not related to genotype D subtypes, neither to donor status, being 77.7% on samples from first-time donors and 74.1% on samples from regular donors (*p* = n.s.), neither to HBV serological patterns. Five of the seven positive samples by HBsAg Next were positive for HBV-DNA by real-time PCR at levels ranging from 1.6 to 11 IU/mL. Of note, one of the samples positive by the HBsAg Next assay showed escape mutations at amino acid positions 121, 123, 134 and 145 (Table 3). A complete summary of testing data is provided in Supplemental Table S1.

## 4. Discussion 

The use of nucleic acid testing (NAT) for hepatitis B virus DNA (HBV-DNA) in the screening of blood donations has been adopted since 2008 in Italy, in addition to screening for HBsAg, to further reduce the risk of transfusion-transmitted HBV [5,16]. The implementation of HBV NAT, especially with the adoption of highly sensitive methods on individual donations (ID-NAT) instead of pooling samples, had the initial aim to unveil HBV infections in the very early stage by reducing the initial ‘window period’ (WP) by 10–20 days compared to the detection of HBsAg [5,13]. After just a few years from the start of HBV NAT, it became apparent that the gain in sensitivity was overwhelmingly due to the detection of occult HBV infections (OBI), i.e., chronic infections characterized by low, often transient, levels of circulating HBV DNA and undetectable HBsAg, with or without.

Other HBV markers (anti-HBc and/or anti-HBs). In a recent review on HBV screening in Italy from 2009 to 2018 on 30,842,794 donations, 1378 donors were found HBV-DNA positive but HBsAg negative and out of those only 3.1% had an acute HBV infection while 96.9% (203 FT and 838 R donors) had an OBI. [17]. Though infectivity of such donations is lower than that from donors in the WP of the acute phase of infection, evidence shows that blood donations collected from OBI donors can transmit HBV to the recipients, and in the aforementioned review [17], the residual risk of transmission by OBI donors was calculated to be approximately 22% higher than that due to NAT WP infections, due to the much higher prevalence of OBI compared to acute hepatitis B. In 2018, Candotti et al. [18] reported HBV transfusion transmission by blood components obtained from donors with an OBI even when they carried a viral load as low as 16 copies and possibly lower, thus near the sensitivity limit of ID-NAT assays. The transmission was more frequent by fresh frozen plasma, was related to the volume transfused and could be prevented by donors being positive for anti-HBs, as previously shown by other authors [19]. More recently, El Ekiaby et al. [20] have reviewed HBV infectivity data and reassessed the 50% infectious dose (ID50) in different HBsAg-positive infection stages, enabling modelling of transfusion-transmitted HBV. By their data, the risk of transmission from HBsAg positive/ID-NAT non-reactive blood transfusion was estimated at 9–46% for components containing 20–200 mL of plasma assuming an ID50 of 316 (point estimate between 100 and 1000) virions, and this has been recently verified by the analysis of seven cases of HBV transmission in Japan [21] where the estimated infectious doses ranged between 2 and 2300 virions. 

The transmission routes of HBV in blood donors with OBI are not easy to ascertain. In our experience, only 54.7% of donors reported any risk factor, much higher than the 24% reported at a national level [17] but still unsatisfactory. Moreover, the relevance of the most frequently reported risk factor (dental care) for HBV infection in blood donors has been recently questioned in a comprehensive review [22]. In our opinion, this apparent poor efficiency of donor selection criteria is due to OBI having been likely acquired many years in the past as an asymptomatic infection that went unrecognized as the pre donation questionnaire only covers risk behaviors occurring over the six months prior to the donation. The relative efficiency of donor selection and the frequency of OBI among regular donors (77.9% of cases in our observation) that may have a history of blood donations spanning several years or even decades, enhance the need for highly sensitive methods for both HBV-DNA and HBsAg. HBV-DNA assays are intrinsically more sensitive than HBsAg assays and finding donations positive for HBV-DNA and negative for HBsAg is quite common [5,17] (5, 17), but the opposite happens with a not negligible frequency. In the Italian survey carried out in 2019 [17] among HBV positive donors, 73.4% were positive for both HBsAg and HBV DNA, 22.1% were positive for HBV DNA and negative for HBsAg, and 4.5% were positive for HBsAg alone, and in a recent experience from China Ye et al. [23] screened 101,025 blood donations and identified 157 (0.16%, 95% confidence interval, 0.13–0.18%) HBsAg ELISA-positive/NAT-negative plasma samples; of those, 71 (45.2%, or 0.07% of total donations) were HBsAg confirmed positive by further HBsAg testing and DNA positive by molecular tests with increased sensitivity. Candotti et al. [24] confirmed the presence of extremely low levels of circulating DNA-containing viral particles in ID-NAT non-reactive or non-repeated reactive blood donations with concomitant high HBsAg levels and anti-HBc reactivity; and in our experience, only five out of seven samples positive for HBsAg by the highly sensitive assay were positive for HBV-DNA by real-time PCR and the median values of HBV-DNA in the samples positive by that assay was 7 IU/mL, confirming the very low replication rates of HBV in OBI cases. These data, along with the evidence of fluctuating patterns of HBV-DNA in OBI [3,13,25], strongly discourage discontinuation of HBsAg in the screening of blood donations [20]. 

Though OBI is an intrinsically milder form of HBV infection, it maintains a definite clinical relevance because, when transmitted, it may cause a classic form of hepatitis B. Moreover, it may reactivate in immunosuppressed or immunocompromised individuals with severe consequences and may contribute to the evolution of liver disease to cirrhosis, also maintaining the oncogenic properties of overt HBV infections [25]. Since testing for HBV-DNA in serum or plasma may not be sufficient to detect all cases of OBI, the potential role of anti-HBc testing is under discussion. While anti-HBc reactivity is considered the most reliable marker of HBV infection at all stages except the very early phase of an acute infection [4,13,26], only <1% of anti-HBc positive first-time donors tested positive for HBV-DNA in Italy [6] and adding anti-HBc to pre-donation screening may reduce unnecessarily the pool of blood donors [16]. On the other hand, in the report from Ye et al. [23] 70/71 donors positive for HBsAg and negative for HBV-DNA at initial screening were positive for anti-HBc and the implementation of anti-HBc screening in Japan has reduced the rate of transfusion-transmitted HBV cases to 0.19 per million donations and OBI-related transmission of HBV was eliminated [21]. On the other hand, persistent anti-HBc-negative OBI should also be considered to mitigate the residual risk of HBV transfusion-transmission. This particular aspect of OBI has been recently characterized and has been reported to account for 8–12% of OBI in Asia [27].

The most interesting finding in our study is the very high frequency of mutations (75%) in the *a* determinant of HBsAg identified in the group of blood donors with OBI. This rate is much higher than reported in other studies on HBV-infected blood donors [26,28,29]. Several factors may explain our findings. First, all donors in our study were infected by HBV genotype D, which is the most prevalent in Italy and in the Mediterranean basin in general [30,31], and genotype D has a high level of heterogenicity both in the S and P (polymerase) genes [32]. Notably, Harris et al. [28] identified mutations in approximately 14% of HBsAg reactive donations from a donor population mostly infected by HBV genotype D, with the most common (46%) at amino acid positions 143 to 145. Other studies on HBV genotype D have demonstrated a high frequency of mutations at amino acid position 120 that were found in 47.2% of our cases, as well as a generally high frequency of mutations in the *a* determinant compared to other genotypes [30]. More recently, an increased rate of mutations in patients infected by genotype D in Italy has been reported [33]. Another factor that may explain a high mutation rate in blood donors with OBI in our study is a long-lasting HBV infection in that population; El Chaar et al. [34] demonstrated that OBIs) have a high frequency of amino acid substitutions in the major hydrophilic region of the S protein and suggested that the increase in escape mutations is a mechanism to evade detection by the host immune system. Moreover, S gene mutations may also impact the detection of hepatitis B surface antigen [33,34], which is already secreted at lower levels in HBV genotype D infection compared to other genotypes [35]. Lastly, a primary infection by mutated HBV strains may also occur in blood donors [36] as well as in their sexual contacts [37], further confirming the potential threat of HBV transmission by donors with OBI. 

The main limitation of our study is a selection bias on the population enrolled, as we limited our observation to OBI donations from which a large enough volume of surplus plasma was available for serology and molecular testing. The sample population studied may therefore not be fully representative of our population of blood donors with an OBI, though the main demographic characteristic (age and gender distribution) was aligned with data observed during that period. Another minor limitation is having been able to test by the Pre-S1/S PCR only on samples from 91 out of 95 donors and perform sequence analysis only on samples from 28 donors due to the very low HBV-DNA load. However, we think that the overall picture would not change much due to the high frequency of mutations in OBI cases reported by several other studies. 

## 5. Conclusions

Our data indicate that HBV infection is still present in Italian volunteer blood donors despite a mass vaccination campaign established more than 30 years ago. This is probably due to the high rate of HBV infection in the pre-vaccination era [12], the silent development of HBV persistence in latent form and the establishment of OBI [26]. Our data show a strikingly high number of mutations in blood donors with OBI: mutations developed without a selective pressure from vaccination, due to age, nor by antiviral therapy—such subjects would not have been accepted as blood donors if diagnosed with chronic hepatitis and treated—and could represent an adaptation of the virus to the host’s immune response, though a recent primary infection by mutated HBV strains cannot be ruled out. The fluctuating pattern of HBV-DNA in OBI suggests that despite the current availability of highly sensitive HBV NAT methods, screening for HBsAg will be maintained and new-generation assays will enhance the detection of low levels of this marker that may be still correlated to infectivity.

## Figures and Tables

**Table 1:** Demographic characteristics of the study population, HBV serological markers, HBV-DNA testing results. FT = first-time donors; R = regular donors; N = number; + or pos = positives; RT = real time; IU = International Units: PCR = polymerase chain reaction.

Donor Group	N	Age (Years, Median)	Anti-HBc & Anti-HBs+	Anti-HBc+	Anti-HBs+	No HBV Marker	HBsAg Next Pos	RT HBV-DNA (%)	HBV-DNA IU/mL (Median)	Pre-S/S PCR Pos/ Tested	Pre-S/S PCR Pos. (%)	S Gene Mutations (%)
FT Females	4	59	1	2	0	1	0	25.0%	35	2/4	50.0%	50.0%
FT Males	17	57	8	7	1	1	2	23.5%	9.1	4/17	23.5%	75.0%
FT Total	21	57	9	9	1	2	2	23.8%	9.6	6/21	28.6%	66.7%
R Females	12	55	7	4	0	1	0	33.3%	6.2	4/11	36.4%	100%
R Males	62	58	31	29	2	0	5	30.6%	6.3	18/59	30.5%	72.2%
R Total	74	58	38	33	2	1	5	31.1%	6.3	22/70	31.4%	77.3%
Females	16	55	8	6	0	2	0	31.3%	7.1	6/15	40.0%	83.3%
Males	79	58	39	36	3	1	7	29.1%	6.9	22/76	28.9%	72.7%
**Total**	**95**	**57**	**47**	**42**	**3**	**3**	**7**	**29.5%**	**7.0**	**28/91**	**30.8%**	**75.0%**

**Table 2:** HBV genotype and S gene mutations on samples from 95 volunteer blood donors positive by Pre-S/S PCR. M = male; F = female; RT = reverse transcriptase.

Donor ID	Gender	Age	HBV Genotype	S-Gene Escape Mutations	Notes
12	M	60	D3	A128V, M133T, Y134N, D144E	4 strains in #12
12	M	60	D3	P120Q, A128V, G130R, T131N, M133T, D144E	4 strains in #12
12	M	60	D3	P120Q, A128V, G130R, T131N, M133T, D144E	4 strains in #12
12	M	60	D3	T126N, Q129R, M133T, C139S, G145A	4 strains in #12
13	M	58	D3	P120S, M133I, D144E	4 strains in #13
13	M	58	D3	P120S, M133I, D144E	4 strains in #13
13	M	58	D3	P120S, M133I, D144E	4 strains in #13
13	M	58	D3	P120S, M133I, D144EG	4 strains in #13
14	M	51	D4	P120A, A128V, Y134H, D144E, G145R	2 strains in #14, one with RT R169H
14	M	51	D4	P120A, A128V, Y134H, D144E, G145R	2 strains in #14, one with RT R169H
17	F	52	D3	P120T, Q129R, M133T	
19	F	42	D4	none	
21	M	55	D3	T118R, P120S, T126I, D144E	
22	M	56	D3	T118K, P120S	
23	M	60	D3	T118K, P120T, A128V, Y134N	
24	M	61	D3	T118K	
27	M	59	D3	none	
33	M	63	D3	none	
37	M	45	D3	120S, 134N	
41	M	58	D3	100C	
45	F	64	D2	P120T, Y134H	
48 *	M	61	D3	118R, 133T	2 strains in #48
48 *	M	61	D3	none	2 strains in #48, RT I169V, V173L
49	M	65	D1	none	
51	F	55	D3	118R	RT I169L
55	M	59	D3	none	
58	M	61	D3	none	
64	M	58	D3	none	
67	F	34	D3	Y134S, P142R, D144E	
68	M	57	D3	C137W	
69	M	60	D3	P120S, C121Y, T123N, T126I, Y134N, D144E	
75	M	59	D4	P120T, R122K, Q129H, M133T	
78	M	69	D3	P120S, S143L	
79	M	57	D3	L109R	
82	F	65	D4	D144A	
96	M	44	D3	121Y, 123N, 134H, 145R	

* Same sample, two different HBV strains.

**Table 3:** Details on the seven samples that tested positive for HBsAg by the HBsAg Next assay. R = regular donor; FT = first-time donor; M = male; S/CO = sample to cutoff ratio; IU = International Units; TND = target not detected; NT = not tested, negative for HBV-DNA by Pre-S1/S PCR.

Donor Group	Gender	Age (Years)	Anti-HBc S/CO	Anti-HBs mIU/mL	HBsAg Next S/CO	HBV-DNA IU/mL	Pre-S1/S PCR Positive/n. Replicates	S-Gene Escape Mutations
R	M	54	5.52	19.7	6.93	TND	0/8	NT
FT	M	50	0.52	0.69	36.96	TND	0/6	NT
R	M	58	7.18	1.13	8.03	31	0/8	NT
R	M	62	6.73	>1000	14.89	4.8	0/4	NT
R	M	43	8.10	0	3.7	6.2	0/10	NT
R	M	59	7.89	31.03	33.17	1.6	0/4	NT
FT	M	44	8.21	0,4	1.3	11	2/18	121Y, 123N, 134H, 145R

## Data Availability

Full data from this study are not available due to confidentiality on some procedures employed for testing samples.

## Supplementary Materials

The following supporting information can be downloaded at: https://www.mdpi.com/article/10.3390/v16010071/s1, Figure S1: S-Gene amino acids 91–179; Table S1: Results of serological and molecular tests for occult HBV infections from 95 blood donors.
