# Supplementary material for: Serological and Molecular Characterization of Occult HBV Infection in Blood Donors from South Italy
Source: Viruses. 2023 Dec 31;16(1):71. doi: 10.3390/v16010071 (PMC10819115; doi:10.3390/v16010071)
Supplement: Supplementary file 1 [file viruses-16-00071-s001.zip › Suppl Fig 1. S-gene aa 91-170 sequence .pdf]

```
                              100       110       120       130       140       150
                     ....|....|....|....|....|....|....|....|....|....|....|....|
Consensus D S-gene   LIFLLVLLDYQGMLPVCPLIPGSSTTSTGPCRTCTTPAQGTSMYPSCCCTKPSDGNCTCI
12-01A               .........SR.I.....PNL...N...VIR...M.AVPN..TN.F...I...E......
12-02A               ..........R.T.......L........QF.....AV.RN.T..F.....T.E......
12-04A               ..........R.T.......L.....N.IQ....M.AV.RN.T..F...NT.E......
12-08A               ..........R.I.....PNL...N....IG....NA.RK..TD.F..S...T.A.....
13-01A               ...................L.........S............I..........E......
13-05B               ...................L.........S...........YI..........E......
13-08B               ...................L.........S...........YI..........E......
13-09A               ...................L.........S...........YI..........E......
13-09B               ...................L.........S...........YI..........X......
14-03A               .........FR..................A.Q....LV.....H.......NTER.....
14-04B               .........FR..................A.Q....LV.....H.......NTER.....
14-05B               .........FR..................A.Q....LV.....H.......NTER.....
14-08B               .........FR..................A.Q....LV.....H.......NXER.....
14-10B               .........FR..................A.Q....LV.....H.......NTER.....
17-A                 ..................I..........T........R...T.................
19-A                 ............................................................
19-B                 ............................................................
21-09A               .V.............M..PL....N..R.S.L...I..P..........I...E......
21-10A               .V.............M..PL....N..R.S.L...I..P..........I...E......
22-01A               .V.............M...L....N..K.S.LP...H......CR....I..........
23-01B               .....G...S..I.R............K.T..D...LVL.K..N................
24-06B               ............I..........L...K................................
24-10A               ............I..........L...K................................
27-01A               ..................TNLT............M.T.......................
27-03A               ...S.A...F........................M.T......X................
27-08A               ..................TNLT............M.T...........F...........
33-A                 .....A.............R.RN.....................................
45-05A               ............I..............A.T......T......H................
45-06B               ............I..............A.T......T......H................
55-A                 .....A......I...............................................
67-02A               .........F....L.............E.......I......S.......R.ET....T
67-07B               .........F....L.............E.......I......S.......R.ET....T
67-11A               .........F....L.............E.......I......S.......R.ET....T
68-04B               ..............................................W.............
69-08B               ..........R.I.......TR.A...PVSY.N..IT......N.F.....H.E......
75-A                 ..........L.TF......L........T.K....L.HA..T.................
79-01A               ..................R.........................................
79-09A               ..................R.........................................
37-03B               .............................S.............N................
41-05A               .........C...........R......K...IL...........YS.............
41-09A               .........C...........R......K...IL...........YS..I..........
48-08A               .T...........F..........N..R......M.V.....XXXXXX............
48-11B               ...............X..TNLT............M.T.......................
49-11A               ............................................................
51-06B               .........S.........L.....V.R........L.......................
58-09A               ..............................R...............F.............
64-02A               ............................................................
64-05B               ............................................................
96-04B               ..........R.................E.Y.N..........H..........R.....
78-9B                ............I..........A.....S......................L.......
78-11B               ............I..........A.....S......................L.......
82-2A                .....................................................A......
```

```
                             160       170
                        ....|....|....|....|
Consensus D S-gene      PIPSSWAFGKFLWEWASARF
12-01A                  H........R..........
12-02A                  .........N....L.....
12-04A                  .........N...D......
12-08A                  .............G......
13-01A                  .................V..
13-05B                  ....................
13-08B                  ....................
13-09A                  ....................
13-09B                  ....................
14-03A                  .........N........H.
14-04B                  .........N........H.
14-05B                  .........N..........
14-08B                  .........N........X.
14-10B                  .........N..........
17-A                    ....................
19-A                    ....................
19-B                    ....................
21-09A                  ....................
21-10A                  ....................
22-01A                  ....................
23-01B                  .........N..........
24-06B                  ....................
24-10A                  ....................
27-01A                  .............A......
27-03A                  ........A...........
27-08A                  .............A......
33-A                    ................L...
45-05A                  ....................
45-06B                  ....................
55-A                    .............G......
67-02A                  ....................
67-07B                  ....................
67-11A                  ....................
68-04B                  ........E...........
69-08B                  ...Q.....R...G......
75-A                    ..............S.....
79-01A                  .................V..
79-09A                  .................V..
37-03B                  ....................
41-05A                  ....................
41-09A                  ....................
48-08A                  ........A...........
48-11B                  .............A......
49-11A                  ....................
51-06B                  L..Q.....N..........
58-09A                  .............V......
64-02A                  ....................
64-05B                  ....................
96-04B                  ....................
78-9B                   ....................
78-11B                  ....................
82-2A                   ....................
```

Supplemental Figure 1. S-gene amino acids 91-170 sequences from OBI samples. Sequence ID contains donor number-replicate identifier. Sequences are aligned to consensus genotype-D sequence; identical to consensus amino acids are shown as dots.
