# Supplementary material for: Serological and Molecular Characterization of Occult HBV Infection in Blood Donors from South Italy
Source: Viruses. 2023 Dec 31;16(1):71. doi: 10.3390/v16010071 (PMC10819115; doi:10.3390/v16010071)
Supplement: Supplementary file 1 [file viruses-16-00071-s001.zip › Supplemental Table 12-1-23 revised.pdf]

Supplemental Table 1. Results of serological and molecular tests for occult HBV infections from 95 blood donors.

Donor ID	*Age at Donation (Years)*	*HBV-DNA QUALIT*	*HBV-DNA QUANT*	*HBsAg*	*HBcAb*	*Donation Date*	*Pos preS1-S PCR, Seq, N+/N PCR reps*	Genotype	S-gene escape mutations	Comments	ARCH HBsAgNx, S/CO	RealTime HBV DNA, 0.5 ml, Log IU/ml	ARCH anti-HBc II 8L44, S/CO	ARCH anti-HBc II Interpretation	ARCH anti-HBs (AUSAB), mIU/mL	ARCH anti-HBs (AUSAB) Interpretation
1	36	POS	NEG	NEG	POS	16.01.2019	0/10	PCR neg			0.3	TND	8.56	Reactive	3.39	Nonreactive
2	56	POS	NEG	NEG	POS	20.03.2019	0/10	PCR neg			0.23	TND	7.52	Reactive	8.29	Grayzone
3	59	POS	NEG	NEG	POS	03.04.2019	0/18	PCR neg			0.29	TND	7.63	Reactive	679.04	Reactive
4	53	POS	NEG	NEG	POS	16.05.2019	0/6	PCR neg			0.26	TND	4.39	Reactive	33.68	Reactive
5	54	POS	<10 UI/ML	NEG	POS	15.06.2019	0/24	PCR neg			0.3	1.11	7.93	Reactive	0.78	Nonreactive
6	44	POS	NEG	NEG	POS	17.07.2019	0/16	PCR neg			0.32	TND	8.15	Reactive	2.35	Nonreactive
7	43	POS	NEG	NEG	POS	07.08.2019	0/18	PCR neg			0.32	TND	6.97	Reactive	4.88	Nonreactive
8	65	POS	NEG	NEG	POS	31.08.2019	0/16	PCR neg			0.26	0.51	4.93	Reactive	0.55	Nonreactive
9	60	POS	NEG	NEG	POS	01.09.2019	0/14	PCR neg			0.25	TND	6.34	Reactive	28.54	Reactive
10	54	POS	NEG	NEG	POS	12.09.2019	0/22	PCR neg			0.28	TND	5.46	Reactive	8.15	Grayzone
11	57	POS	NEG	NEG	POS	01.10.2019	0/22	PCR neg			0.26	TND	6.01	Reactive	385.78	Reactive
12	60	POS	<10 UI/ML	NEG	POS	06.10.2019	4/20	D3	A128V, M133T, Y134N, D144E	4 strains in #12	0.27	0.98	7.35	Reactive	1.35	Nonreactive
12	60	POS	<10 UI/ML	NEG	POS	06.10.2019	4/20	D3	P120Q, A128V, G130R, T131N, M133T, D144E	4 strains in #12	0.27	0.98	7.35	Reactive	1.35	Nonreactive
12	60	POS	<10 UI/ML	NEG	POS	06.10.2019	4/20	D3	P120Q, A128V, G130R, T131N, M133T, D144E	4 strains in #12	0.27	0.98	7.35	Reactive	1.35	Nonreactive
12	60	POS	<10 UI/ML	NEG	POS	06.10.2019	4/20	D3	T126N, Q129R, M133T, C139S, G145A	4 strains in #12	0.27	0.98	7.35	Reactive	1.35	Nonreactive
13	58	POS	NEG	NEG	POS	20.10.2019	5/20	D3	P120S, M133I, D144E	4 strains in #13	0.29	TND	2.61	Reactive	0.76	Nonreactive
13	58	POS	NEG	NEG	POS	20.10.2019	5/20	D3	P120S, M133I, D144E	4 strains in #13	0.29	TND	2.61	Reactive	0.76	Nonreactive
13	58	POS	NEG	NEG	POS	20.10.2019	5/20	D3	P120S, M133I, D144E	4 strains in #13	0.29	TND	2.61	Reactive	0.76	Nonreactive
13	58	POS	NEG	NEG	POS	20.10.2019	5/20	D3	P120S, M133I, D144EG	4 strains in #13	0.29	TND	2.61	Reactive	0.76	Nonreactive
14	51	POS	<10 UI/ML	NEG	POS	03.11.2019	5/22	D4	P120A, A128V, Y134H, D144E, G145R	2 strains, one with RT R169H	0.26	1.01	6.35	Reactive	90.94	Reactive
14	51	POS	<10 UI/ML	NEG	POS	03.11.2019	5/22	D4	P120A, A128V, Y134H, D144E, G145R	2 strains, one with RT R169H	0.26	1.01	6.35	Reactive	90.94	Reactive
15	59	POS	NEG	NEG	POS	09.11.2019	0/4	PCR neg			0.26	TND	6.14	Reactive	80.67	Reactive
16	67	POS	<10 UI/ML	NEG	POS	17.11.2019	0/20	PCR neg			0.27	TND	5.54	Reactive	30.38	Reactive
17	52	POS	<10 UI/ML	NEG	POS	22.11.2019	1/2	D3	P120T, Q129R, M133T		0.29	0.06	7.57	Reactive	1.48	Nonreactive
18	50	POS	NEG	NEG	POS	28.12.2019	0/12	PCR neg			0.23	TND	7.59	Reactive	58.2	Reactive
19	42	POS	2,55 10E1	NEG	POS	02.11.2017	2/2	D4	none		0.31	1.55	0.07	Nonreactive	0.31	Nonreactive
20	56	POS	<10 UI/ML	NEG	POS	23.02.2017	0/6	PCR neg			0.28	0.71	6.87	Reactive	0.87	Nonreactive
21	55	POS	NEG	NEG	POS	08.04.2017	2/22	D3	T118R, P120S, T126I, D144E		0.26	0.52	7.8	Reactive	5.1	Nonreactive
22	56	POS	NEG	NEG	POS	04.08.2017	1/20	D3	T118K, P120S		0.27	0.3	7.54	Reactive	4.07	Nonreactive
23	60	POS	NEG	NEG	POS	04.10.2017	1/26	D3	T118K, P120T, A128V, Y134N		0.25	TND	7.85	Reactive	22.79	Reactive
24	61	POS	NEG	NEG	POS	07.09.2017	2/22	D3	T118K		0.3	0.5	7.43	Reactive	6.19	Nonreactive
25	65	POS	<10 UI/ML	NEG	POS	19.09.2017	0/20	PCR neg			0.3	1	6.4	Reactive	15.68	Reactive
26	63	POS	NEG	NEG	POS	07.11.2017	0/22	PCR neg			0.3	TND	4.88	Reactive	6.69	Nonreactive
27	59	POS	NEG	NEG	POS	14.11.2017	3/12	D3	none		0.68	TND	8.38	Reactive	0	Nonreactive
28	57	POS	NEG	NEG	POS	28.07.2016	0/8	PCR neg			0.38	TND	4.98	Reactive	74.41	Reactive
29	54	POS	NEG	NEG	POS	07.08.2016	0/8	PCR neg			6.93/6.62/ 6.51	TND	5.52	Reactive	19.7	Reactive
30	50	POS	NEG	NEG	POS	13.08.2016	0/6	PCR neg			36.96/37.23/37.1	TND	0.52	Nonreactive	0.69	Nonreactive
31	58	POS	<10 UI/ML	NEG	POS	27.08.2016	0/8	PCR neg			8.03/8.35/8.66	1.49	7.18	Reactive	1.13	Nonreactive
32	62	POS	<10 UI/ML	NEG	POS	25.08.2016	0/4	PCR neg			14.89/13.95/14.29	0.68	6.73	Reactive	> 1000.00	Reactive
33	63	POS	<10 UI/ML	NEG	POS	11.01.2018	1/2	D3	none		0.25	0.67	8.19	Reactive	50.25	Reactive
34	51	POS	NEG	NEG	POS	22.01.2018	0/8	PCR neg			0.29	TND	6.2	Reactive	36.87	Reactive
35	68	POS	NEG	NEG	POS	06.02.2018	0/10	PCR neg			0.25	TND	5.62	Reactive	62.17	Reactive
37	45	POS	<10 UI/ML	NEG	POS	13.03.2018	1/6	D3	120S, 134N		0.26	TND	2.05	Reactive	0.71	Nonreactive
38	43	POS		NEG	POS	13.06.2018	0/10	PCR neg			3.7/3.72/3.46	0.79	8.1	Reactive	0	Nonreactive
39	50	POS		NEG	POS	20.06.2018	0/8	PCR neg			0.34	TND	5.57	Reactive	4.87	Nonreactive
40	48	POS		NEG	POS	05.11.2018	Insufficient volume				0.25	TND	6.97	Reactive	9.33	Grayzone
41	58	POS		NEG	POS	20.11.2018	2/18	D3	100C		0.27	TND	7.23	Reactive	> 1000.00	Reactive
43	59	POS	NEG	NEG	POS	26.12.2018	0/4	PCR neg			33.17/31.69/31.03	0.2	7.89	Reactive	31.03	Reactive
44	65	POS		NEG	POS	24.03.2019	0/14	PCR neg			0.28	TND	6.92	Reactive	79.33	Reactive
45	64	POS		NEG	POS	08.12.2018	2/22	D2	P120T, Y134H		0.3	0.85	7.39	Reactive	0.15	Nonreactive
46	50	POS	NEG	NEG	POS	25.05.2020	Insufficient volume				0.24	TND	7.82	Reactive	24.02	Reactive
47	50	POS	NEG	NEG	POS	17.05.2020	0/22	PCR neg			0.24	TND	8.05	Reactive	16.58	Reactive
48	61	POS	<10 UI/ML	NEG	POS	20.06.2020	2/22	D3	118R, 133T	2 strains, 1 w/o escape mutations a	0.25	TND	8.55	Reactive	0	Nonreactive
49	65	POS	NEG	NEG	POS	18.05.2020	1/24	D1	none	s W74*	0.23	TND	4.33	Reactive	4.45	Nonreactive
50	64	POS	NEG	NEG	POS	24.04.2020	0/20	PCR neg			0.26	TND	5.79	Reactive	36.94	Reactive

Donor ID	Age at Donation (Years)	HBV-DNA QUALIT	HBV-DNA QUANT	HBsAg	HBcAb	Donation Date	Pos preS1-S PCR, Seq, N+/N PCR reps	Genotype	S-gene escape mutations	Comments	ARCH HBsAgNx, S/CO	RealTime HBV DNA, 0.5 ml, Log IU/ml	ARCH anti-HBc II 8L44, S/CO	ARCH anti-HBc II Interpretation	ARCH anti-HBs (AUSAB), mIU/mL	ARCH anti-HBs (AUSAB) Interpretation
51	55	POS	NEG	NEG	POS	14.03.2020	1/22	D3	118R	RT I169L	0.26	TND	7.03	Reactive	18.7	Reactive
52	55	POS	NEG	NEG	POS	14.06.2020	0/22	PCR neg			0.24	TND	7.38	Reactive	70.77	Reactive
53	60	POS	<10 UI/ML	NEG	POS	25.10.2011	0/5	PCR neg			0.25	1.33	6.94	Reactive	10.89	Grayzone
54	58	POS	<10 UI/ML	NEG	POS	23.07.2020	0/8	PCR neg			0.23	1.09	6.06	Reactive	24.25	Reactive
55	59	POS	<10 UI/ML	NEG	POS	22.07.2020	1/2	D3	none		0.24	1.17	7.22	Reactive	1.16	Nonreactive
57	57	POS		NEG	POS	02.08.2020	0/4	PCR neg			0.28	TND	7.56	Reactive	10.84	Grayzone
58	61	POS		NEG	POS	16.08.2020	1/20	D3	none		0.24	TND	0.97	Nonreactive	98.2	Reactive
59	68	POS		NEG	POS	16.08.2020	0/18	PCR neg			0.29	TND	8.13	Reactive	2.13	Nonreactive
60	66	POS		NEG	POS	26.08.2020	0/20	PCR neg			0.26	TND	6.34	Reactive	202.03	Reactive
61	51	POS		NEG	POS	28.08.2020	0/18	PCR neg			0.25	TND	1.57	Reactive	2.93	Nonreactive
62	51	POS		NEG	POS	16.08.2020	0/18	PCR neg			0.23	TND	2.26	Reactive	7.88	Nonreactive
63	58	POS		NEG	POS	16.08.2020	0/20	PCR neg			0.27	TND	4.75	Reactive	10.07	Grayzone
64	58	POS		NEG	POS	25.08.2020	2/16	D3	none		0.54	TND	8.19	Reactive	6.85	Nonreactive
65	45	POS		NEG	POS	28.08.2020	0/18	PCR neg			0.22	TND	3.51	Reactive	16.56	Reactive
66	55	POS		NEG	POS	06.09.2020	0/12	PCR neg			0.26	TND	5.87	Reactive	0.36	Nonreactive
67	34	POS		NEG	POS	06.09.2020	3/26	D3	Y134S, P142R, D144E		0.31	0.72	3.92	Reactive	6.97	Nonreactive
68	57	POS		NEG	POS	10.10.2020	1/18	D3	C137W		0.32	1.16	6.59	Reactive	27.45	Reactive
69	60	POS		NEG	POS	29.08.2020	1/18	D3	P120S, C121Y, T123N, T126I, Y134N, D144E		0.26	0.93	7.75	Reactive	2.14	Nonreactive
70	61	POS		NEG	POS	03.10.2020	0/12	PCR neg			0.25	TND	6.22	Reactive	48.45	Reactive
71	59	POS		NEG	POS	13.10.2020	0/12	PCR neg			0.25	TND	3.27	Reactive	47.3	Reactive
72	50	POS		NEG	POS	18.10.2020	0/12	PCR neg			0.31	TND	0.77	Nonreactive	3.8	Nonreactive
74	62	POS		NEG	POS	25.10.2020	0/6	PCR neg			0.25	TND	4.27	Reactive	77.68	Reactive
75	59	POS		NEG	POS	29.07.2020	1/2	D4	P120T,R122K, Q129H, M133T		0.3	0.72	7.55	Reactive	24.25	Reactive
76	56	POS		NEG	POS	17.06.2020	0/10	PCR neg			0.24	TND	5.95	Reactive	5.07	Nonreactive
77	61	POS		NEG	POS	03.10.2020	0/8	PCR neg			0.28	TND	6.38	Reactive	49.26	Reactive
78	69	POS		NEG	POS	21.11.2020	2/24	D3	P120S, S143L		0.3	TND	6.59	Reactive	0.51	Nonreactive
79	57	POS		NEG	POS	29.11.2020	2/26	D3	L109R		0.23	1.07	5.58	Reactive	16.4	Reactive
80	55	POS		NEG	POS	19.12.2020	0/18	PCR neg			0.25	0.84	8.23	Reactive	13.3	Reactive
81	56	POS		NEG	POS	14.02.2021	0/4	PCR neg			0.68	TND	8.51	Reactive	4.56	Nonreactive
82	65	POS		NEG	POS	13.12.2020	1/28	D4	D144A		0.25	TND	4.66	Reactive	15.7	Reactive
83	55	POS		NEG	POS	13.12.2020	0/24	PCR neg			0.23	TND	7.17	Reactive	14.42	Reactive
84	62	POS		NEG	POS	18.12.2020	0/4	PCR neg			0.25	TND	6.32	Reactive	9.89	Grayzone
85	60	POS		NEG	POS	28.12.2020	0/2	PCR neg			0.21	TND	0.08	Nonreactive	24.77	Reactive
86	70	POS		NEG	POS	29.12.2020	0/24	PCR neg			0.25	TND	3.31	Reactive	585.34	Reactive
87	56	POS		NEG	POS	29.12.2020	0/20	PCR neg			0.31	TND	0.06	Nonreactive	14.18	Reactive
88	69	POS		NEG	POS	03.01.2021	0/20	PCR neg			0.27	TND	6.91	Reactive	0.64	Nonreactive
89	42	POS		NEG	POS	17.01.2021	0/14	PCR neg			0.22	TND	3.73	Reactive	5.18	Nonreactive
90	54	POS		NEG	POS	31.01.2021	0/2	PCR neg			0.18	TND	7.76	Reactive	5.15	Nonreactive
91	38	POS		NEG	POS	06.02.2021	Insufficient volume				0.18	TND	7.78	Reactive	2.16	Nonreactive
92	54	POS		NEG	POS	08.11.2020	0/22	PCR neg			0.21	TND	2.03	Reactive	3.3	Nonreactive
93	59	POS		NEG	POS	15.11.2020	0/22	PCR neg			0.31	TND	7.06	Reactive	13.05	Reactive
94	62	POS		NEG	POS	15.11.2020	0/24	PCR neg			0.27	TND	7.1	Reactive	22.87	Reactive
95	62	POS		NEG	POS	03.12.2020	0/22	PCR neg			0.26	0.8	6.95	Reactive	12.58	Reactive
96	44	POS		NEG	POS	08.12.2020	2/18	D3	121Y, 123N, 134H, 145R		1.3/1.3/1.31	1.04	8.21	Reactive	0.4	Nonreactive
97	52	POS		NEG	POS	06.12.2020	0/20	PCR neg			0.33	TND	7.34	Reactive	> 1000.00	Reactive
98	56	POS		NEG	POS	25.02.2021	Insufficient volume				0.23	TND	7.29	Reactive	45.26	Reactive
99	63	POS		NEG	POS	02.03.2021	0/6	PCR neg			0.21	TND	6.44	Reactive	31.15	Reactive
